# “#I-Am-Engaged”: Conceptualization and First Implementation of a Multi-Actor Participatory, Co-designed Social Media Campaign to Raise Italians Citizens’ Engagement in Preventing the Spread of COVID-19 Virus

**DOI:** 10.3389/fpsyg.2020.567101

**Published:** 2020-11-05

**Authors:** Guendalina Graffigna, Caterina Bosio, Mariarosaria Savarese, Marina Barello, Serena Barello

**Affiliations:** ^1^EngageMinds HUB – Consumer, Food and Health Engagement Research Center, Università Cattolica del Sacro Cuore, Milan, Italy; ^2^Department of Psychology, Università Cattolica del Sacro Cuore, Milan, Italy; ^3^Faculty of Agricultural, Nutrition and Environmental Sciences, Università Cattolica del Sacro Cuore, Milan, Italy

**Keywords:** COVID-19, behavioral change, health engagement, communication campaign, prevention, Patient Health Engagement Model, health communication

## Abstract

The COVID-19 pandemic forced health authorities around the world to introduce public health measures to contain the risks of contagion. This greatly impacted on citizens’ quality of life, often raising concerns and reactance. There is an ongoing urgent need to promote and sustain behavioral changes and adherence to preventive measures. Based on the theoretical framework of the Patient Health Engagement Model and a participatory co-design process, a social media campaign aimed at improving citizens’ health engagement toward behavioral change for preventing the spread of COVID-19 was promoted in Italy in the early months of the pandemic. In this paper, we describe the methodological process adopted to develop the campaign, its characteristics, and the first results—in terms of audience reach and engagement in its early implementation. The discussion of this grounded-up and citizen-centered approach to social campaign development highlights key ways of promoting learning, engaging citizens, and supporting their participation in the co-production of educational interventions for behavioral change toward preventive actions.

## Introduction

On January 30, 2020, the World Health Organization (WHO) declared the Coronavirus epidemic a public health emergency of international concern (Duff, 2020). In little more than a month, starting on March 9, 2020, the entire country of Italy was forced into lockdown. In order to contain the COVID-19 epidemic, government authorities took extreme measures, such as the closure of cities and regions, the closure of schools and offices, the reorganization of health services, the restriction of transportation, and stopping people from leaving home except for urgent needs (Saglietto et al., 2020). This introduced new challenges that the country was poorly prepared to handle. As the numbers of cases rapidly increased, there was growing evidence that behavioral changes were required for citizens to reduce the risks of transmission. For this reason, large scale public health communication interventions were implemented to raise citizens’ awareness, and responsibility, increasing their literacy about the restrictive measures (Bonell et al., 2020).^1^ The decision took into account the lessons learned from the management of previous epidemic experiences such as HIV, SARS, Ebola (Vijaya et al., 2004; Smith, 2006; Figueroa et al., 2014; Friedman et al., 2016; Gillespie et al., 2016; Figueroa, 2017; Rose, 2017; Bedson et al., 2019). However, there are still problems of poor adherence to such measures (Briscese et al., 2020; McFadden et al., 2020; van Rooij et al., 2020). As in the management of previous epidemic experiences, the initial unavailability of an effective drug therapy/vaccination had forced the authorities to activate “non-pharmacological” interventions of a social nature, fostering behavioral change to mitigate the impact of the pandemic by leveraging the capability of citizens to adhere to the preventive public health measures (Ferguson et al., 2006; Godoy et al., 2012). Furthermore, people and community engagement during public health emergencies have been increasingly recognized as an important component to enable behavioral changes to reduce the spread of disease (Schoch-Spana et al., 2007; Bedson et al., 2019). The role of social media through an educational campaign was extensively examined using the scientific literature and was a key element in promoting behavioral change (Agha, 2003; French et al., 2010; Denecke and Atique, 2016; Fayoyin, 2016).

Empowering communities during the emergency phase and improving their psychological motivation to adhere to restrictive measures, norms and regulations was critical due to the feeling of uncertainty that can undermine the “psychological commitment” of people when adopting new life rules: not only in the acute phase of the emergency but also, and above all, in the medium-long term management of the epidemic (Sniehotta et al., 2005). As a long tradition of scientific studies in psychology has shown, enhancing preventive behavioral change is a long and challenging process (Forkan et al., 2015). Adherence to the new measures could be represented as a roller-coaster: a bumpy journey with moments in which citizens feel motivated to adhere and moments in which fatigue and frustration prevail causing them to renounce new habits (Rubin et al., 2009). It is challenging to monitor and sustain psychological engagement and the motivation to change behaviors, and it is even more crucial that people do so in the COVID-19 era (van Bavel et al., 2020).

In this paper, we describe the process of conceiving developing, and first launching of a public educational campaign (named “#I-am-engaged”) aimed at sustaining Italian citizens’ engagement and adherence to the COVID-19 preventive measures, with the ultimate aim to create changes that will psychologically endure people, enabling people to cope with the long term impact of the pandemic.

### The Theoretical Framework: The Patient Health Engagement Model

Many models of behavioral change have been developed over the years, trying to identify the factors which may support or inhibit the adoption of preventive behaviors. For instance, the Health Belief Model (Maiman and Becker, 1977) and the Protection Motivation Theory (Rogers and Prentice-Dunn, 1997) have shown that risk avoidance depends on an individual’s beliefs about their susceptibility to the risk and the perceived severity of the health threat. This is connected to the perceived effectiveness of actions that will avoid risk and the individual’s self-confidence in their ability to perform them. The Theory of Planned Behaviors (Ajzen, 2011) and its further developed form (the Integrative Model of Behavioral Prediction) (Yzer, 2012) that aims to magnify the role of social norms in the process.

Although these models are effective in orienting educational campaigns for behavioral change, they lack a full considering of the role of emotional dynamics in that process. Recent studies have underlined the importance of considering the role of anticipated emotions and desires in predicting the intention to change in health behaviors by proposing the integration of the Theory of Planned Behaviors (Perugini and Bagozzi, 2001; Kim et al., 2013). Prochaska and DiClemente used a Transtheoretical model of change that further emphasized the role of process-like emotional dynamics by underlining the role of sub-conscious determinants of an individuals’ motivation to change health behaviors (Prochaska and DiClemente, 2005). Building on these arguments, by integrating the lesson learned from the studies on the process of griefs and griefing and the conceptualization of the Five Stage of Loss model by Kubler-Ross (1969), we elaborated the Patient Health Engagement Model (PHE-Model) (Graffigna and Barello, 2018) with the ambition of describing the subjective emotional dynamics which undermine changes to health behaviors. Different from the previously mentioned models, the PHE-Model points to the crucial role of psychological willingness to engage in health risk prevention. The PHE-Model describes the process of the emotional and motivational reframing of an individuals’ role in perception in the management of a health risk condition and its consequences, evolving from being a passive user of services to an active partner of the healthcare system and healthcare professionals (Barello et al., 2020). The model describes four psychological positions on a continuum from minimum to maximum engagement: “Blackout” (complete disengagement, psychologically freezing and behaviorally paralysis), “Arousal” (initial awareness of the risky situation but lack of skills to manage it effectively), “Adhesion” (effective emotional regulation and coping with the risk condition), “Eudaimonic Process” (ability to deal with the uncertainty of the moment and a strong motivation to become proactive and responsible for personal health behaviors) (Graffigna et al., 2017a). In particular, the highest position of the PHE-Model (namely, Eudaimonic Project) depicts a psychological condition of full consciousness about people one’s role and responsibility in the management of their health, thanks to a positive approach to life and illness, to the ability to correctly navigate (i.e., find and use) health information, to an effective adjustment to hazard to health conditions, and to an ability to cope with the uncertainty of the situation and the related psychological distress (Barello and Graffigna, 2015; Graffigna and Barello, 2018). Therefore, the Eudaimonic Project status, which is the higher level of engagement described by the PHE model, requires people to reframe on both emotional and cognitive-behavioral levels (Menichetti et al., 2018).

Previous studies conducted on different patient populations have demonstrated that a high level of engagement, as measured with the PHE-Model, is predictive of a higher adherence to medical prescriptions and a better-informed search for online information (Graffigna et al., 2017a). The assumption behind the application of the PHE-Model to a “non-patient” population relies on its psychological nature. The PHE-Model considers health engagement as the function of an emotional process of elaboration and adaptation to a critical health event. This critical health event in the case of chronic patients often coincides with clinical diagnosis, or with a new symptom, or with a new request for a life style change for medical reasons. In the case of the COVID19 emergency, the risk of contagion from Sars-Cov-2 is—at the psychological level—an analog critical health event that can trigger the psychological ability of individuals (patients and not) to cope with risk and to engage in changes to health behavior. To prove this concept in relation to the recent COVID-19 pandemic, a high level of PHE is predictive of a safer adaptation to the emergency, considering different targets of the general population, such as adults and students (Graffigna et al., 2020; Nania et al., 2020).

Based on these considerations, we adopted the PHE-Model as a theoretical framework for orienting the first conceptualization and development of the *#I-am-Engaged* campaign, starting from the hypothesis that public adherence to preventive measures during a pandemic emergency requires a deep consideration of the moods and emotions of citizens: monitoring and fostering individuals’ positive emotional elaboration of a critical event—such as the COVID-19 pandemic—as a function of the psychological readiness to engaged in their health protection, a situation which is crucial to sustaining people’s ability to preventively cope with COVID19.

### Context of the Campaign

The concept and the design of the campaign *#I-am-Engaged* were based on three subsequent methodological steps:

1. A quantitative cross-sectional online survey on a representative sample of the Italian population aimed at investigating the psychological impact of the COVID-19 pandemic, the level of Italian citizens’ engagement in the COVID-19 preventative measures, and their attitudes toward media and informative sources on the topic.
2. A participatory design approach to define contents, tone of voice, modes and the media mix of the educational campaign.
3. A preliminary analysis of the first output of the campaign launch in terms of audience reach.

More in detail, the three phases can be methodologically described as follows.

## Materials and Methods

### The Cross-Sectional Online Survey

Before developing the campaign and its content, an online survey of a representative sample of Italian citizens was conducted between February 28 and March 4, 2020, to understand people’s reactions to the COVID-19 pandemic’s outbreak and collect insights about their unmet needs for behavioral change, which could be addressed by the social campaign.

The study took place between February 28 and March 4, 2020. A sample of 1,000 Italians, who were representative of the Italian population for gender, age, employment, geographical area, and from the urban centers of residence in all the different regions of Italy. Participants were over 18 years old and completed a self-administered online questionnaire. The sample was recruited through a random selection from the consumers’ panel managed by Norstat srl. The eligibility criteria for being involved in the study were that all participants had to aged 18 years or older, being able to read and understand Italian, and live in Italy.

After recruitment and informed consent, responders were asked to complete an online survey involving questions about health engagement, affective response, and behavioral responses to the COVID-19 pandemic. The full methodological details and results of this survey are reported in an extensive paper currently being submitted (Graffigna et al., 2020).

### The Participatory Design of the Campaign

The study involved a participatory process in which researchers, representatives of patients organizations as well as clinicians were facilitated and actively participated in designing the aims, contents, and format of the social campaign, based on their personal representations, meanings, priorities, and needs (Charania and Tsuji, 2012). The stakeholders involved in the participatory process were selected based on their previous experience in promoting the engagement of individuals toward the management of their health, as attested by previous publications on the topic, participation in educational initiatives, and/or active engagement in patient advocacy campaigns. In particular, we involved representatives of Patients’ Organizations as they are the bearers of a chronic experience that challenges them daily with are engaged in adopting appropriate behaviors for effective health management. They are an example of psychological engagement with behavioral change and of persistence in the adherence to prescribed changes in lifestyle due to disease management (see Table 1 for details).

**TABLE 1:** Stakeholders of “I am engaged” campaign.

Expert’s category	Number of experts involved in the campaign generation
Patient associations’ representatives	16
Health care professionals	4
Researchers	6

The campaign’s participatory design process followed these steps: first, the results of the survey were presented, discussed, and enriched by a wide community of communication experts, patient engagement advocates, and laypeople using a live Facebook webinar. The strategy, target, content, tone of voice of the campaign aimed at promoting citizens’ engagement in behavioral change was co-designed with a group of stakeholders using an iterative email process of discussion and sharing starting from the first draft proposed by the research team. Improvement was suggested by the stakeholders in further steps of reconfiguration until the final version of the campaign structure and contents was achieved.

### First Proof of Concept: Audience Reach and Engagement

Evaluating the impact of social communication campaigns is always hard due to the many intervening factors that can influence a real-world setting (Bloom, 1980; White, 2014; Veríssimo et al., 2018). Due to the explorative nature of the #I-am-engaged campaign, and the time constraints in developing it, due to the COVID19 emergency, we were not able to structure a systematic process of evaluation for its effectiveness. This limit is typical of social communication campaigns launched during a critical event (Firestone et al., 2017). However, in order to provide preliminary proof of concept for our campaign, we monitored the first quantitative data of audience-reach and engagement in the first period of launch (from March 10, to May 27, 2020). The following indicators (which are commonly considered for social media marketing campaigns, e.g., Barger and Labrecque, 2013; Hair et al., 2017) were considered: number of views (only for Facebook live videos), number of likes, number of comments, number of people reached and number of shares. These indicators were collected with the metrics released from Facebook and LinkedIn platforms.

## Results

### Main Findings From the Online Survey

The survey study involved a total of 976 participants with a mean age of 44 years (*SD* = 14; range 18–70). Of the 1,000 citizens contacted, 24 reported missing data in the questionnaire and were excluded from the analysis. For a more detailed description of the study (see Table 2).

**TABLE 2:** Demographic profiles of the sample involved in the online survey (*N* = 968).

	n	%		n	%
**Gender**			**Chronic patient**		
Male	473	48.9	Yes	174	18.0
Female	495	51.1	No	794	82.0
**Age**			**Geographical area**		
18–24	99	10.1	North-West	253	26.0
25–34	156	16.1	North-East	178	18.4
35–44	209	21.6	Center	194	20
45–54	215	22.2	South and Islands	343	35.4
55–59	106	11.0			
60–70	183	19.0			
**Education**			**Coming from “red zones”**		
Middle school or lower	142	14.6	Yes	294	30.3
High school	586	60.6	No	674	69.7
University degree	240	24.8			
**Employment**			**Inhabited center size**		
Laborer	203	20.9	Up to 5,000 inhabitants	163	16.8
Office worker	153	15.8	5/10,000 inhabitants	150	15.5
Unoccupied	147	15.2	10/30,000 inhabitants	241	24.9
Housewife/man	146	15.1	30/100,000 inhabitants	189	19.5
Freelance professional	119	12.3	100/500,000 inhabitants	102	10.6
Retired	76	7.9	More than 500,000 inhabitants	117	12.1
Student	53	5.5	Missing	6	0.6
Manager	36	3.7			
Teacher	18	1.8			
Other	17	1.8			

Regarding the engagement variable, only 16% of our sample resulted in a higher position (i.e., “Eudaimonic project,” with no significant differences between genders, or among age ranges (see Table 3).

**TABLE 3:** Percentage of engagement levels in the overall sample and across different demographic groups.

	PHE model positions			
	
	Blackout	Alert	Adhesion	Eudaimonic project
Overall sample (%)	1.1	21.4	61.5	16
**Gender (%)**	χ^2^(*df* = 3, *n* = 968) = 9.122; *p* = 0.028			
Male	1.3	18.2	61.7	18.9
Female	1.0	24.4	61.2	13.3
**Age (%)**	Fisher’s exact *p*-value = 0.139, based on 10,000			
	Montecarlo’s simulations			
18–24	0	16.5	64.9	18.6
25–34	0.6	25	63.5	10.9
35–44	2.4	25.2	57.1	15.2
45–54	1.4	23.7	59.5	15.3
55–59	0.9	18.7	65.4	15.0
60–70	0.5	15.2	62.5	21.7

*Pearson’s Chi-squared and Fisher’s Exact test were used to compare distributions.*

When asked to report on the attitudes toward preventive behaviors required to mitigate the contagion spread, a small portion of the sample was asked to adhere to measures such as “Bought a face protective mask” (13.2%), “Canceled travels abroad” (25.3%), Reduced daily movements whenever possible (39.8%) (see Table 4). Regarding people’s use of information and literacy about COVID-19 preventive measures, our sample reported that they used more than one source of information and were, on average, highly literate about the required preventive behaviors (see Table 5).

**TABLE 4:** Citizens’ attitudes toward preventive behaviors to mitigate the COVID-19 spread.

Preventive behaviors	% of compliant
Canceled travel abroad	25.3
Increased hands washing	78.0
Reduced meals out	33.3
Bought a face protective mask	13.2
Avoid getting close to influenced people	71.4
Avoid crowded places	67.4
Reduced daily movements whenever possible	39.8

**Preventive attitudes (1 = Disagree, 5 = agree)**	**M (SD)**

I am the most responsible in preventing the contagion by COVID-19	3.74 (0.92)
Preventive behaviors for COVID-19 are an act of social responsibility	4.16 (0.86)
I dedicate much time in getting informed about health	3.45 (0.85)
I usually share with my General Practitioner concerns regarding my health status	3.31 (1.0)
I am used to telling my General Practitioner unusual symptoms regarding my health	3.47 (0.86)

**TABLE 5:** Citizens’ reported use of sources of information and literacy regarding COVID-19.

Frequency of use of source of information (1 = Never; 5 = more than once a day)	M (SD)
Newscast	3.82 (1.04)
Television programs	3.06 (1.18)
Radio	2.57 (1.23)
Websites	3.14 (1.24)
Social networks	2.77 (1.37)
Specialized magazines	1.87 (1.11)
Newspapers	2.37 (1.25)
Scientific journals	1.87 (1.12)
General practitioner	2.13 (1.16)
COVID-19 info phone number	1.42 (0.93)

**Literacy on COVID-19**	**% of correct responses**

Are there specific medicines for the treatment or prevention of COVID-19?	89.7
Does COVID-19 hit both young and old people?	93.2
Does the vaccine against pneumonia protect against COVID-19?	91.8
Do pets transmit COVID-19?	91.6
Is it safe to receive a mail or a package from an area with a high rate of infected people?	78.9
Spraying alcohol or chlorine on the body may kill the new coronavirus?	84.3
Are antibiotics useful in preventing the infection by the new coronavirus?	88.9

### “#I-Am-Engaged” Campaign: Conceptual Structure and First Implementation

**FIGURE 1:**
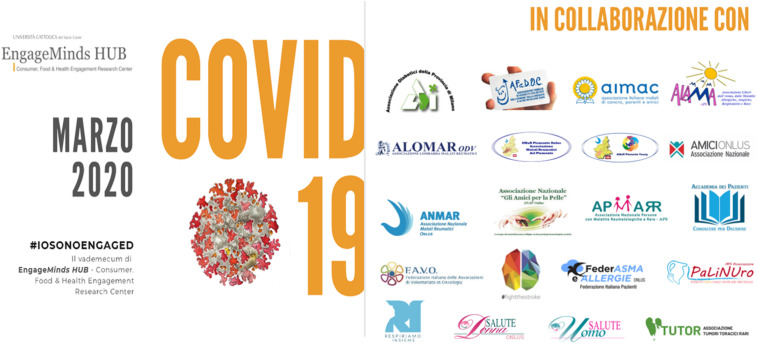
The cover and last pages of the Vademecum—(we have received the appropriate permissions from the copyright holder of this figure to publish it).

We conducted round tables with researchers and relevant stakeholders about the survey results, which revealed the need and relevance of a public education campaign, targeting adult Italian citizens, to improve the engagement of the population in the management of COVID. In particular, the survey revealed the need to target the motivational levers at the base of people’s engagement in behavioral change, as data showed that people were informed but poor at adapting and changing their habits. This supported the decision to launch the #I-am-engaged campaign project. To reach the final configuration of the campaign, we conducted 34 rounds of telematic interaction. Finally, the panel of experts agreed on the definition of the two main components of the campaign: (1) a Vademecum (see Appendix 1 and Figure 1 for the cover of this document) inspired by the Patient Health Engagement Model and aimed at fostering psychological motivations to engage in more responsible health prevention for COVID19 epidemic, and (2) a Facebook campaign to support the dissemination of such principles effectively and simply.

#### The Vademecum

The Vademecum is a leaflet in which the main contents of the campaign are showcased. The contents included recommendations that were derived from the key theoretical concepts of the PHE-model, and in particular, protocols previously conceptualized, developed, and piloted by the research team (Menichetti and Graffigna, 2016; Guida et al., 2019).

In particular, 10 recommendations were selected and described, anchored to key words that were inspired by the (Italian) acronym of the word engagement: empathize, navigate the right information, manage stress, trust the healthcare system, enjoy the time, be enthusiastic, monitor, balance, new normality, and drag (see Table 6 for a deeper description of its scientific rationale).

**TABLE 6:** The campaign rationale.

Vademecum keyword	Rationale	Conceptual link to the PHE model dimensions	Theoretical roots
Empathize (in Italian: *Empatizza*)	The acceptance of daily life limitations (such as lockdown) required to mitigate the contagion spread requires the individual ability to move from an individualistic consideration of personal benefit to the consciousness of one’s behaviors impact on societal health. This is also a function of an adequate ability to empathize with the other, their needs, and expectations.	Emotional dimension	Barello and Graffigna, 2020; Harper et al., 2020
Navigate the right information (in Italian: *Naviga le corrette informazioni*)	The ability to navigate the corrected information about the virus, to recognize reliable sources of information is a crucial component of health engagement and foremost important in such an emergency such as the one of COVID19	Cognitive-behavioral dimension	Koh et al., 2013; Smith et al., 2013; Palumbo et al., 2016
Deal with stressors (in Italian: *Gestisci lo stress*)	The outbreak of coronavirus disease 2019 (COVID-19) may be stressful for people. Fear and anxiety about the risk of contagion can be overwhelming and cause strong emotions that might reduce people’s engagement in preventive behaviors. Thus, finding strategies to prevent fear and anxiety from turning into distress can help us regain control of our lives, increasing our capacity to respond positively and reducing the anxiety and distress caused by uncertainty in a rapidly evolving situation.	Emotional dimension	Moos, 1992; Daubenmier et al., 2007; Gruman et al., 2010
Trust the healthcare system (in Italian: *Affidati al sistema sanitario*)	Mastering the consciousness of one own role in the healthcare system is a prerequisite of health engagement. Citizens are claimed to perceive themselves not only mere end-users of the healthcare system but also active players for the effectiveness and sustainability of the system. This in particular in the case of a Public National System such as the Italian one	Cognitive-behavioral	Gilson, 2003; Richards et al., 2013; Gabay, 2015
Enjoy time (in Italian: *Gustati il tempo*)	If one disease can provide wisdom beyond our comprehension of how fragile, interconnected and precious life is, the novel coronavirus (COVID-19) pandemic offers citizens a plethora of lessons on the relevance to take time for themselves to engage in a psychological recovery during and after the pandemic. It’s important that people take breaks, and set up an accountability system for their lifestyle. In this situation, people need to ration their time wisely so that they can still meet targets while having a healthy work-life balance.	Emotional	Hunt and Macleod, 1987; Boekaerts, 1992
Be enthusiastic (in Italian: *Entusiasmati*)	It’s easy to let negative thoughts and feelings creep in during the COVID-19 pandemic. Despite all of this, keeping a positive mindset can go a long way in managing through a difficult time. Having an attitude that looks for the positive and tries to be optimistic can help people to filter out some of the constant barrage of bad or discouraging news that might impact on people’s motivation to cope with the difficulties and engagement in healthy behaviors.	Emotional	Meyers and Meyers, 2003; Henley and Donovan, 2004
Monitor yourself (in Italian: *Monitorati*)	Making people able to engage in self-monitor signals and symptoms is critical to assess if they may be at risk for disease and whether they qualify for additional testing or treatment. Symptom progression can occur rapidly and ensuring these people know when and how to seek hospital care can potentially save lives. Engaging people in monitoring and updating their health, medication, or treatment plans have the potential to increase treatment concordance, as well as enabling health care providers to review and intervene if needed.	Cognitive-behavioral	Dolan Mullen et al., 1997; Burke et al., 2002
Keep balance (in Italian: *Equilibrio*)	Balance or balancing served many important purposes in the context of health and illness literature. Achieving balance as a state is recognized as a way to enable people to experience a sense of health and well-being. Research also associated balance with resilience, describing it as a means of coping, gaining inner strength, moderating vulnerability, and adjusting to difficult changes. Balance also helps people to deal with uncertainty, unfamiliarity, and unpredictability. Balance or balancing is also a source of consolation that could help people deal with adversity. Balancing seemed to improve people’s emotional experiences and self-esteem because it provided the necessary stability to prioritize commitments, helped people to resolve ambivalence, provided people with confidence about decisions made, and reduced guilt about value conflicts.	Emotional	Mullen, 1992; Lipworth et al., 2011
Find a new normality (in Italian: *Nuova normalità*)	New understanding about people’s role in their health management and getting engaged in preventive behaviors calls attention to recognizing new forms of adaptations and new habits that encourage people’s own coping and creative processes to deal with their strain and, in some cases, reconstruct everyday lives.	Cognitive-behavioral	de Ridder et al., 2008; Darcy et al., 2014
Be a leader of change (in Italian: *Trascina*)	Making a difference through guiding others, building awareness, and sensitizing the enlarged community is recognized as a characteristic of people fully engaged in their health. Helping others to adopt recommended behaviors to enable a virtuous circle of “health engagement contagion.”	Cognitive-behavioral	Stanhope and Henwood, 2014; Anderson and McCleary, 2015

All the recommendations of the Vademecum were explained with plain and simple text and developed in an extended graphic document uploaded on the university website and broadly virtualized by the Facebook campaign described below. The creative development of the Educational Campaign has aimed at facilitating the transmission and the understanding of these messages, as well as sharing and ensuring adhesion to the new measures. Specifically, on a stylistic level, we considered: (1) tone of voice: adoption of a concise, concrete, simple and immediate language; (2) visual style: the COVID molecule was chosen as the campaign’s identifying icon for all messages, and graphically reconfigured in a non-medical, pleasant and reassuring style (watercolor, soft shades); and (3) reputational reinforcement was supported by the fact that all the stakeholders put logos on the campaign materials.

#### The Campaign First Implementation

The Facebook Campaign included the following actions:

• Hashtag: we created the hashtag #I-am-engaged (in Italian: #Io-sono-engaged) as an anchor/reference for all the messages and interactions of the campaign.
• Facebook posts: The Vademecum was shared step by step (one keyword with the associated recommendation at a time) through three posts per week on EMH social pages and broadly shared by all the stakeholders involved in the participatory co-design.
• Facebook live videos: The dissemination of the Vademecum was supported and made more dynamic by a few Facebook live videos aimed at deepening the contents of the Vademecum and increasing awareness of the campaign. From a stylistic point of view, we opted for short live videos (30 min), highly interactive (thanks to the real-time exchange of opinions through the comments on the Facebook platform) and aimed at building a “ritual/usual” appointment, reproducing the normality of everyday life and closeness among people (consistently the name of the live broadcasts was “A coffee with EngageMinds HUB”). At least 10 appointments have been planned but this planning depends on the evolution of the pandemic.
• Video testimonies: Furthermore, the development of the campaign included a re-launch of content by encouraging video testimonies from followers of the campaign. The theme of the testimonies was sharing personal experiences and concrete engagement actions to manage the health emergency effectively. The videos were made in the form of short messages (30 s) and featured the same graphic references to characterize the entire campaign. The collected testimonies were shared twice a week on EngageMinds HUB social pages.

### Proof of Concept: Preliminary Results on Audience Reach and Engagement

The campaign was launched on March 10, 2020, and was still ongoing at the time of this article’s publication. The Vademecum was released completely, the Facebook live videos are in progress, as is the collection and dissemination of video testimonies. The total release of these contents is scheduled for the coming months. However, it is anticipated that the campaign will be adapted in response to its progress, based on the evolution of the experience of longer-term ‘cohabitation’ with COVID19, which is difficult to predict at this time.

The results were updated on May 27, 2020, and are summarized in Tables 7, 8. Overall, these preliminary data show that the campaign was able to reach more than 40.000 people (33.390 on Fb, 12.689 on Ln). Although it is not possible to exclude the duplicated reach of the two channels, these results appear relevant and promising if compared with the whole Italian population. Potentially, the campaign reached one-third of the Italian population. Out of this, only 10% of the audience showed an active engagement with the campaign, by expressing likes (697 on Fb, 311 on Ln), by writing comments (102 on Fb, 10 on Ln), or sharing its contents (253 on Fb, data not available for Ln). Furthermore, these data show how audience reach and engagement was higher on Facebook than LinkedIn, probably due to the different nature of these platforms and their different targeted audiences. Facebook generally appears a more suitable platform for engaging with the audience and as a means to convey public health information in a lively manner.

**TABLE 7:** The results of the campaign—Facebook metrics.

	N	People reached	Views (of Facebook lives)	Interactions	Like	Sharing	Comments
Post	57	33,390		2,652	697	253	102
Facebook live videos	6		3,346				
Video testimonies	16						

**TABLE 8:** The results of the campaign—LinkedIn metrics.

	N	Views	Like	Comments
Post	47	12689	311	10
Video testimonies	16			

## Discussion

The COVID19 pandemic has demonstrated how the behaviors of individuals are crucial to prevent contagion risks both for individual citizens and the whole community. In the absence of vaccination, the availability of pharmaceutical treatment still uncertain, and behavioral rules such as physical distancing, wearing face masks, and other hygiene norms will be crucial in containing the spread of the virus (Hellewell et al., 2020). This requires a huge change in people’s attitudes toward health prevention and their understanding of the crucial role they play in sustaining the healthcare system’s ability to face the emergency. The engagement of individuals in this process, in becoming more aware of their role in health prevention, is regarded as a key aim for public health authorities across the world. However, so far, the majority of public health campaigns, particularly in Italy, have mainly focused on transferring literacy about the virus and behaviors (Crosier et al., 2015). Sustaining people’s awareness and education toward COVID-19 health prevention requires a more complex approach and several combined actions (van Bavel et al., 2020). In particular, emotional reactions to the fear of contagion are important drivers of people’s behaviors during a pandemic (Kim and Niederdeppe, 2013). The levels of the perceived threat to one’s health are often related to an increased avoidance of health risks, but only when subjects are also equipped with the right literacy and skills (Maiman and Becker, 1977). Furthermore, as time passes and people become used to the emergency, it is important to orient educational initiatives to profoundly change people’s attitudes toward health prevention. In the long-term, the concept of health engagement becomes crucial to ensure people’s ability not only to acquire knowledge about the virus but also to become conscious about their responsibility in preventing the contagion for themselves and their community (Sniehotta et al., 2005). However, fostering the psychological readiness of individuals and encouraging them to engage in health prevention requires the development of initiatives aimed at supporting them in their emotional regulation and positive adaptation to stress and uncertainty of the emergency (Cameron and Leventhal, 2012). People need to promote a critical attitude toward the correct navigation of health information and to acquire competences related to self-monitoring and the self-management of health behaviors (Hibbard and Greene, 2013).

Based on these considerations, this paper illustrates the conception, design, and launch of a social media educational campaign aimed at sustaining more responsible COVID-19 prevention in Italian citizens, entitled the *#I-am-engaged* campaign. The core method of communication adopted in the campaign was a Vademecum, encouraging engagement in healthy habits, based on the Patient Health Engagement Model (Graffigna and Barello, 2018). The engagement principles included in the Vademecum were disseminated digitally via a Facebook campaign including Facebook educational posts, Facebook live videos, video testimonies.

Taking into account recommendations coming from scientific literature, the *#I-am-engaged-campaign* is theoretically anchored to the PHE Model, which identifies engagement as the fundamental leverage for behavioral change, promoting a multi-dimensional activation at a cognitive, emotional, and behavioral level (Graffigna and Barello, 2018). The campaign is also constructed around a community-based perspective, with a participatory process that favors co-creation among peers. Furthermore, the campaign adopts a positive tone of voice focusing on the promotion of good practices. The campaign takes into account the lesson learned in previous communication interventions reported in the literature. The *#I-am-engaged* campaign addresses the following trigger points to enhance people’s engagement in COVID-19 prevention.

(a) *Theory driven approach*: Previous literature has demonstrated that it is crucial to not only focus on the technical aspects of communication, but also have a theoretical frame for understanding health behaviors as drivers to change (Gynther et al., 2012). From this perspective, our campaign used a comparative analysis of behavioral change models applied to health prevention and considered the adoption of these as part of the rationale and psychological model of engagement. This was the basis for selecting the key elements and concepts of the Vademecum in supporting behavioral change.

(b) *Positive tone*: A broad spectrum of psychological evidence has demonstrated how a positive communicative approach is more effective than a “scary” one that emphasizes the negative consequences of risk behavior. A reassuring and empowering tone, focusing on solutions is preferable in the case of health emergencies such as the COVID-19 pandemic, since this can foster individual self-efficacy and a positive attitude toward health prevention. This principle was a key element of the communication strategy adopted by our campaign. Several of the key psychological concepts promoted in the Vademecum have been inspired by Positive Phycology, which is one of the theoretical underpinnings of the Patient Health Engagement Model (Graffigna and Barello, 2018). Furthermore, the overall tone of voice of the campaign is positive, as was also suggested by the stakeholders in the co-design process, and aimed at simplifying and making accessible to everyone the psychological principles of the Vademecum. Finally, the graphic choices of the campaign (a watercolor drawn virus with clear and warm colors) were coherent, aiming to pass a scientific concept in a simplified and positive manner.

(c) *People oriented messages*: Embedding public communication with a deep understanding of the population’s values and attitudes toward health prevention and concerns are fundamental to generating understandable messages to which people will listen (Setbon and Raude, 2009; Gray et al., 2012; Crosier et al., 2015; van Bavel et al., 2020). According to this principle, a crucial step for the development of our campaign was the survey of a representative sample of the Italian population during the first phase of the emergency, which aimed at exploring people’s literacy, attitudes, and levels of engagement toward COVID-19 prevention. The survey, which is discussed in more detail in another paper (Graffigna et al., 2020), confirmed the opportunity to change of people’s attitudes toward prevention, rather than only to increase their literacy. Furthermore, it demonstrated the role of engagement in improving people’s attitudes toward preventive measures during the COVID-19 emergency. The survey also provided the basis for enhancing stakeholder’s discussion about evidence and nurturing the participative co-design process of the campaign.

(d) *Participatory approach*: Another important element for successful health communication is the adoption of a participatory approach that enhances the activation of the target. A prescriptive “medical” and logical approach to preventive education can be ineffective in promoting behavioral change and a “top-down” passage of preventive information, from an expert to laypeople results in poor engagement (Warren, 2004; Crosier et al., 2015). It may also raise psychological resistance and reactance in the target population (Bigi, 2016). Being conscious of this communication risk, we configured the #*I-am-engaged-campaign* as an “engaging campaign” inspired by the concept of people’s participation in health prevention and aiming to foster individual psychological engagement. The campaign adopted a participative co-design during its development, involving key experts and stakeholders in health engagement promotion to ensure that the communicative style and tone of voice aligned with the cultural and social context. This enabled the construction of a solid base of collaboration for the dissemination of the campaign. In particular, the different stakeholders were involved both in patronizing the initiatives and in spreading them to their networks, but also in contributing with video testimonials aimed at making daily prevention the health engagement principles more concrete and applicable. Finally, the Facebook campaign disseminated the Vademecum principles (still in progress) and also aimed to reach further stakeholders and the general public through the hashtag *#I-am-engaged*.

Although the campaign is still ongoing at the time of this submission and social media feedback about its launch is partial, there have been some interesting achievements in terms of audience reach. In addition to the high number of people reached and the levels of social media engagement we achieved on Facebook and LinkedIn, other indicators of success were: (a) the mention of the campaign in the newsletter of Regione Lombardia;^2^ (b) the mention of the Engagement Vademecum and related campaigns among the inspiring principles of the “Seven Steps” guidelines, launched by the Higher Ministry Of Health in Italy;^3^ and (c) the interest of an important media partner in Italy (Radio24, in its program “Obiettivo Salute”)^4^ who partnered with the research team to adapt the campaign and Vademecum principles for radio.

There were also several limitations to this study. First, no data on effectiveness are provided for the campaign. This, however, is a common limitation of social marketing campaigns, launched under the pressure of an emergency to sensitize and inform audiences. Evaluating the impact of social marketing aimed at sustaining changes in health behavior is methodologically challenging due to the many intervening factors that can influence outcomes in a real world environment (Firestone et al., 2017; Veríssimo et al., 2018). Further data regarding the impact of our campaign will be collected in the coming months, both in terms of audience reach and engagement, and in terms of qualitative feedback and levels of appreciation. Due to the current contingency measures related to the pandemic, a structured pre-post evaluation of the campaign’s impact on the audience’s behaviors was not possible. However, future data and feedback about likes from the audience will be important for optimization and personalization, targeting specific population groups (e.g., young people, senior citizens, and so forth). Another potential limitation of the campaign is that it delivers a generalized message aimed at a preliminary sensitization of the population about the importance of engagement. The campaign was based on the analysis of the Italian situation and it will also be necessary to evaluate the transferability of the campaign to other countries characterized by different socio-cultural settings and health policies in the management of COVID-19.

Apart from these limitations, the case of the *#I-am-engaged* campaign is valuable in its conceptual and participatory structure and might potentially contribute to promoting public sensitization and awareness about COVID-19 prevention. The campaign appears to be a particularly valuable way to use social media platforms to foster exchange and Facebook campaigns shared and created a dialogue between scientists and the lay public about topics relevant to the COVID-19 emergency. This developmental process and its key features are potentially innovative and helpful when facing viral emergencies such as COVID-19.

## Data Availability Statement

The raw data supporting the conclusions of this article will be made available by the authors, without undue reservation, to any qualified researcher.

## Ethics Statement

The studies involving human participants were reviewed and approved by the Ethical Commission – Department of Psychology, Università Cattolica del Sacro Cuore Milano. The patients/participants provided their written informed consent to participate in this study.

## Author Contributions

GG conceived the manuscript structure, supervised the project and campaign development, and drafted the methodology. GG and CB drafted the background, the description of the campaign, and the discussion. SB drafted the survey results. MS and MB drafted the analysis of the first launch of the campaign and contributed to the campaign project as a whole. All authors revised the text and approved the final version.

## Conflict of Interest

The authors declare that the research was conducted in the absence of any commercial or financial relationships that could be construed as a potential conflict of interest.

## Footnotes

1 See for example the following institutional campaigns promoted by the National Health Authority of Italy in the first phase of the COVID19 pandemic in Italy https://www.who.int/campaigns/connecting-the-world-to-combat-coronavirus; https://www.health.gov.au/resources/collections/coronavirus-covid-19-campaign -resources; http://www.salute.gov.it/portale/nuovocoronavirus/dettaglioNotizie NuovoCoronavirus.jsp?lingua=italiano&menu=notizie&p=dalministero&id=4221.

2 https://www.promozionesalute.regione.lombardia.it/wps/portal/site/promozione -salute/dettaglioredazionale/temi/stili-di-vita/buone-pratiche-resto-acasa

3 http://www.salute.gov.it/portale/nuovocoronavirus/dettaglioNotizieNuovoCoro navirus.jsp?lingua=italiano&menu=notizie&p=dalministero&id=4689

4 https://www.radio24.ilsole24ore.com/programmi/obiettivo-salute
