# Supplementary material for: “#I-Am-Engaged”: Conceptualization and First Implementation of a Multi-Actor Participatory, Co-designed Social Media Campaign to Raise Italians Citizens’ Engagement in Preventing the Spread of COVID-19 Virus
Source: Front Psychol. 2020 Nov 5;11:567101. doi: 10.3389/fpsyg.2020.567101 (PMC7674954; doi:10.3389/fpsyg.2020.567101)
Supplement: Supplementary file 1 [file Data_Sheet_1.PDF]

UNIVERSITÀ CATTOLICA del Sacro Cuore

EngageMinds HUB

Consumer, Food & Health Engagement Research Center

MARZO 2020

COVID 19

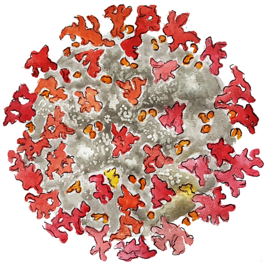

#IOSONOENGAGED

Il vademecum di **Engage*Minds* *HUB*** - Consumer, Food & Health Engagement Research Center

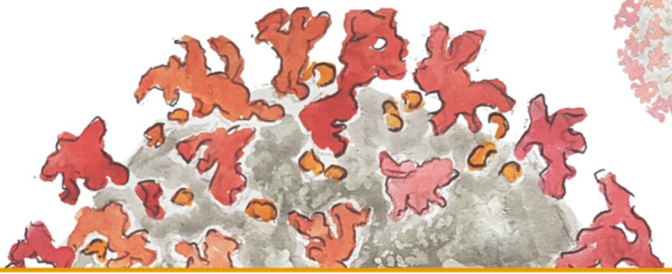

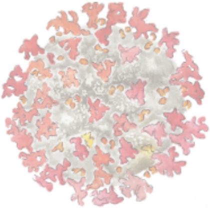

#VADEMECUM D1

UNIVERSITÀ CATTOLICA del Sacro Cuore

EngageMinds HUB

Consumer, Food & Health Engagement Research Center

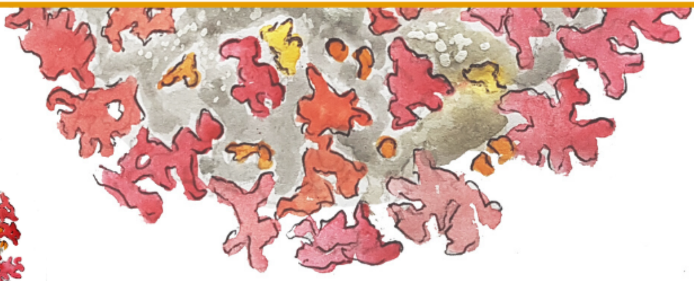

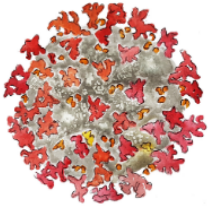

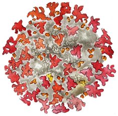

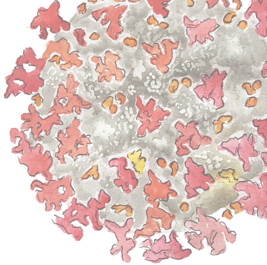

UNIVERSITÀ CATTOLICA del Sacro Cuore

EngageMinds HUB

Consumer, Food & Health Engagement Research Center

COVID-19 ha causato una vera e propria **emergenza sanitaria** e un senso di grande incertezza sul futuro: tutti noi cittadini siamo chiamati a un forte atto di responsabilità individuale e sociale.
Cambiando le nostre abitudini e i nostri comportamenti possiamo dare un fondamentale contributo per limitare la diffusione del CORONAVIRUS.
Si tratta di un momento in cui siamo tutti chiamati a essere “**engaged**”: cioè coinvolti attivamente e responsabilmente nelle attività di prevenzione.
In questo quadro **EngageMinds HUB - Consumer, Food & Health Engagement Research Center** vuole dare il suo contributo condividendo un *vademecum* per affrontare insieme, con piccoli spunti e riflessioni, questo periodo così delicato e di emergenza.
Proponiamo alcune indicazioni utili per gestire al meglio la normale sensazione di incertezza e preoccupazione che ognuno di noi può trovarsi a sperimentare in un momento in cui ci sentiamo fortemente preoccupati per la nostra salute e limitati nelle nostre abitudini quotidiane.
In questo opuscolo sono presenti alcune “parole chiave” che costituiscono dei semplici spunti di riflessione e consigli da mettere in pratica nella nostra vita quotidiana per trovare una nuova forma di normalità.
Sperimentiamo un nuovo modo di essere responsabili nella prevenzione e nella gestione della nostra salute.

#IOSONOENGAGED

UNIVERSITÀ CATTOLICA del Sacro Cuore

EngageMinds HUB

Consumer, Food & Health Engagement Research Center

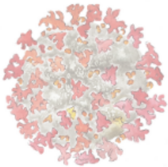

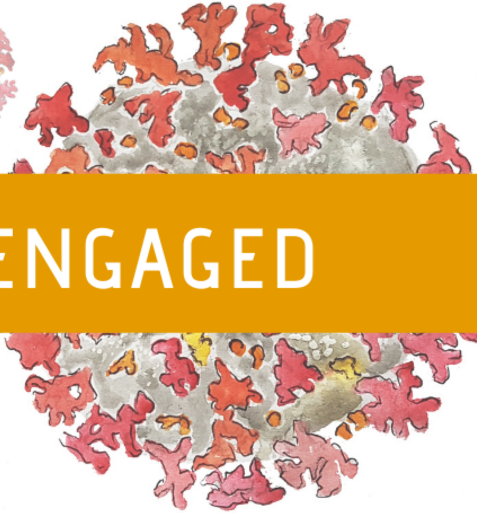

#10 SONO ENGAGED

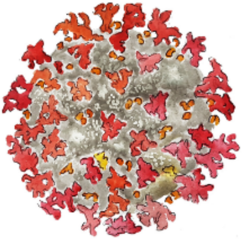

UNIVERSITÀ CATTOLICA del Sacro Cuore

EngageMinds HUB

Consumer, Food & Health Engagement Research Center

Oggi più che mai è fondamentale che i cittadini diventino in prima persona responsabili del loro ruolo nel mitigare gli effetti della diffusione del coronavirus, collaborando con il Sistema Sanitario e facendo la loro parte.

Cosa significa essere #engaged?

#IOSONOENGAGED significa **considerare con attenzione e seguire le indicazioni forniteci dagli esperti** al fine di **tutelare noi stessi e gli altri**, **sentendoci protagonisti del cambiamento** e diventando **buoni esempi** per le persone intorno a noi.
Essere engaged significa **mettere a frutto le nostre risorse e capacità**.

Significa inoltre utilizzare le nostre **emozioni** in modo consapevole al fine di ri-progettare la nostra vita quotidiana in modo soddisfacente, pur nella difficoltà.

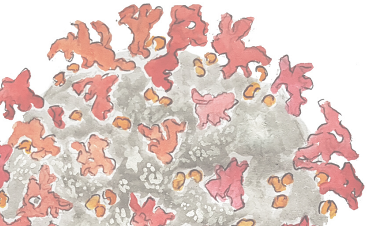

#IOSONOENGAGED

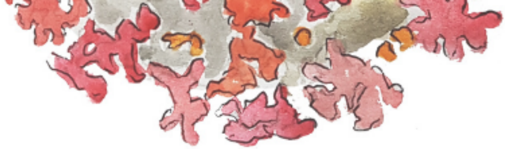

Empatizza
Naviga le giuste informazioni
Gestisci lo stress
Affidati al Sistema Sanitario
Gustati il tempo
Entusiasmati
Monitora
Equilibrio
Nuova normalità
Trascina

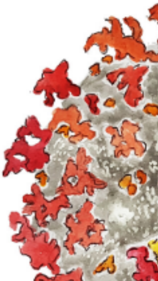

UNIVERSITÀ CATTOLICA del Sacro Cuore

EngageMinds HUB

Consumer, Food & Health Engagement Research Center

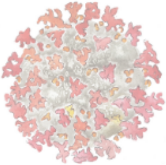

#EMPATIZZA

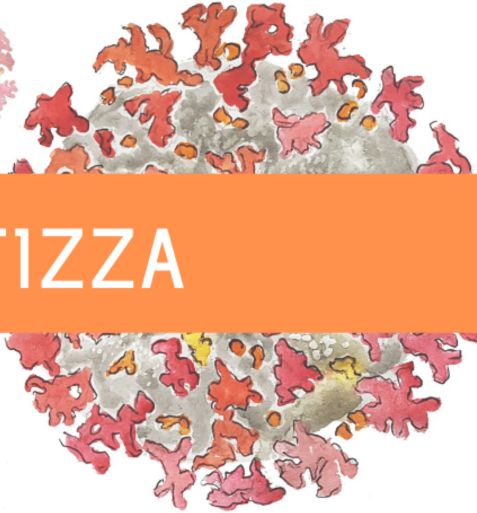

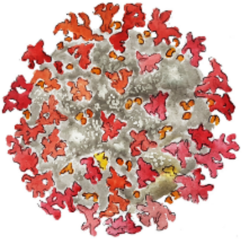

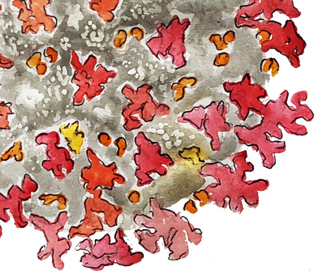

UNIVERSITÀ CATTOLICA del Sacro Cuore

EngageMinds HUB

Consumer, Food & Health Engagement Research Center

Nei momenti difficili abbiamo l'opportunità di tirare fuori il meglio di noi per fare squadra al fine di tutelare noi stessi e gli altri.

Il coronavirus è un banco di prova, un'occasione per ritrovare quel senso di **appartenenza alla nostra comunità** e di **comprensione reciproca** che non solo può preservarci dal contagio, ma può anche salvare vite.

Compiamo un gesto di solidarietà mettendoci nei panni altrui.

#IOSONOENGAGED

UNIVERSITÀ CATTOLICA del Sacro Cuore

EngageMinds HUB

Consumer, Food & Health Engagement Research Center

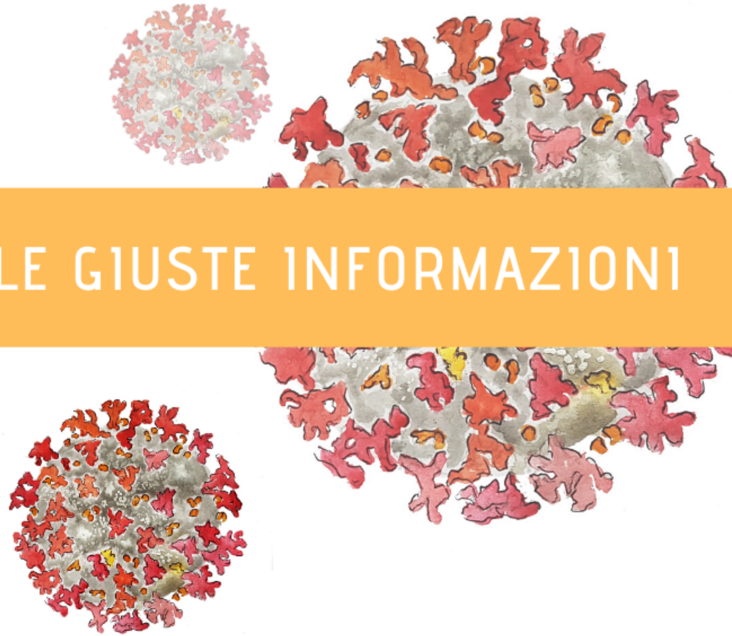

#NAVIGA LE GIUSTE INFORMAZIONI

UNIVERSITÀ CATTOLICA del Sacro Cuore

EngageMinds HUB

Consumer, Food & Health Engagement Research Center

In seguito al diffondersi dell'epidemia da **covid-19**, abbiamo assistito ad una vera e propria **infodemia**: siamo continuamente esposti a informazioni e aggiornamenti non sempre veritieri.

È per questo importante raccogliere informazioni affidabili utilizzando fonti ufficiali come il sito internet **World Health Organization (WHO),** del **Ministero della Salute,** dell'**Assessorato alla Salute** o dell'**Istituto Superiore di Sanità**.

#IOSONOENGAGED

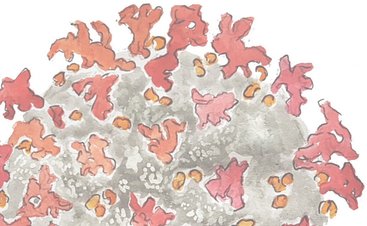

UNIVERSITÀ CATTOLICA del Sacro Cuore

EngageMinds HUB

Consumer, Food & Health Engagement Research Center

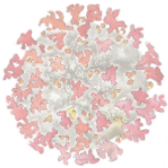

#GESTISCI LO STRESS

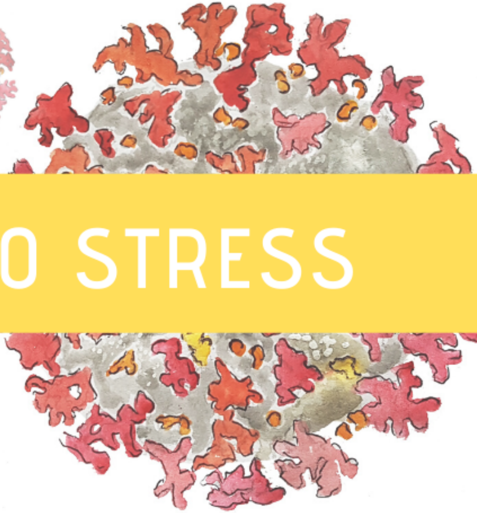

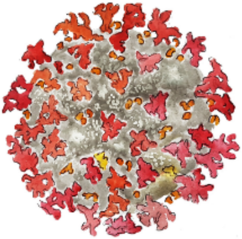

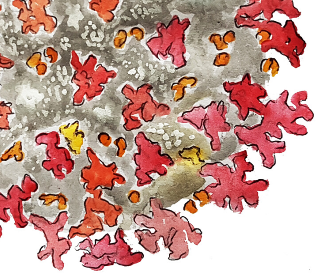

UNIVERSITÀ CATTOLICA del Sacro Cuore

EngageMinds HUB

Consumer, Food & Health Engagement Research Center

Il coronavirus ci dimostra non soltanto di essere un'emergenza sanitaria, ma anche un'epidemia di **insicurezza**, di **paure**, di **ansia** e **stress**.
Si tratta di reazioni umane tipiche di periodi di incertezza.

Il problema è che queste emozioni possono portarci a compiere azioni irrazionali e rischiose per la salute nostra e dei nostri cari.

Ma come possiamo gestirle al meglio?

Condividere queste preoccupazioni con i propri cari, fare cose che ti fanno sentire bene, concederti di realizzare un piccolo sogno o desiderio per cui non hai mai il tempo, possono essere utili suggerimenti.
Se utile, **rivolgiti ad uno psicologo o ad un altro operatore della salute** per **ricevere un supporto specializzato.**

Sentirsi **protagonisti della propria salute** e parte di una comunità che agisce per uno stesso obiettivo aiuta a trovare un **nuovo obiettivo di cambiamento personale** in un momento in cui risulta difficile e gestire lo stress.

#IOSONOENGAGED

UNIVERSITÀ CATTOLICA del Sacro Cuore

EngageMinds HUB

Consumer, Food & Health Engagement Research Center

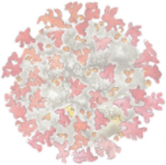

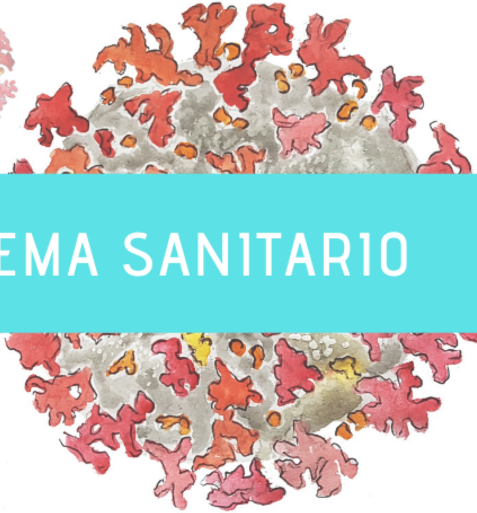

#AFFIDATI AL SISTEMA SANITARIO

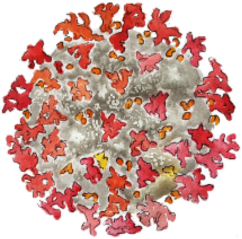

UNIVERSITÀ CATTOLICA del Sacro Cuore

EngageMinds HUB

Consumer, Food & Health Engagement Research Center

Ogni giorno assistiamo ad appelli del personale sanitario a rispettare le misure previste dalle autorità al fine di contenere al massimo il rischio di contagio, per poter garantire le cure necessarie a tutti coloro che presentano dei sintomi.

Per questo è fondamentale che tutti i cittadini si fidino e si affidino alle indicazioni comunicate puntualmente dalle istituzioni poiché costituiscono una condizione imprescindibile per uscire dalla crisi.

Essere ingaggiati significa anche **avere fiducia negli esperti** e **collaborare con il sistema sanitario** per raggiungere gli obiettivi comuni.

#IOSONOENGAGED

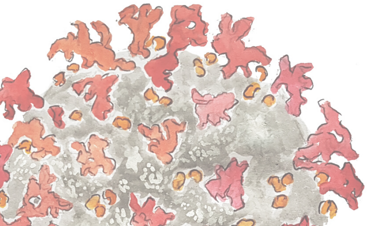

UNIVERSITÀ CATTOLICA del Sacro Cuore

EngageMinds HUB

Consumer, Food & Health Engagement Research Center

#GUSTATI IL TEMPO

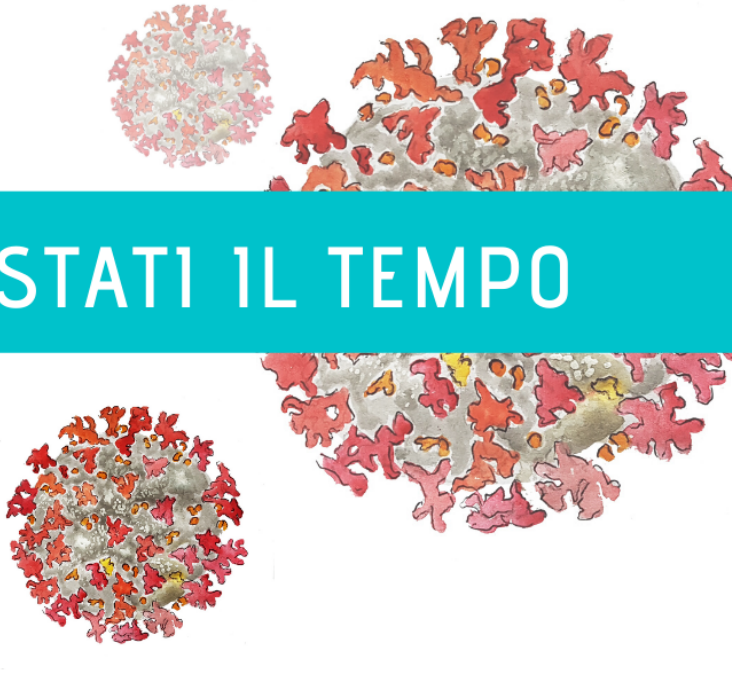

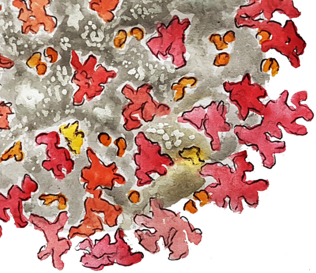

Per il nostro Paese è il momento di mostrare coraggio: aspettare con responsabilità che passi la fase critica e allo stesso tempo elaborare una ripresa, pensare e progettare al meglio il nostro futuro.
Ci siamo **sempre lamentati della mancanza di tempo**, nelle nostre vite frenetiche. **Ora, ciò che non manca, è proprio il tempo**.
Guardiamoci dentro ora perché poche volte nella vita ci capiterà di poter avere del tempo per concentrarsi su noi stessi: scegliamo il modo migliore per ripartire.

Approfittiamone per **riappropriarci delle bellezze della nostra quotidianità**.
Approfittiamo oggi del silenzio, un luogo bellissimo che ci permette di riflettere.
Sognamo il domani quando la chiacchierata a «tu per tu» sarà ciò che ci arricchirà.
Ma soprattutto sfruttiamo questo stop forzato per c**omprendere quanto ogni nostro gesto quotidiano abbia un impatto importante sulla nostra salute**, **sulla salute della collettività e su quella del nostro Paese**.

Prenditi tempo per rimettere a posto i **valori** per cui vale la pena spendere le nostre energie e il nostro tempo.
Prendi l'occasione per **darti nuovi obiettivi di vita e di salute**.

#IOSONOENGAGED

UNIVERSITÀ CATTOLICA del Sacro Cuore

EngageMinds HUB

Consumer, Food & Health Engagement Research Center

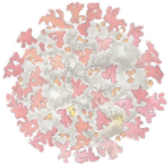

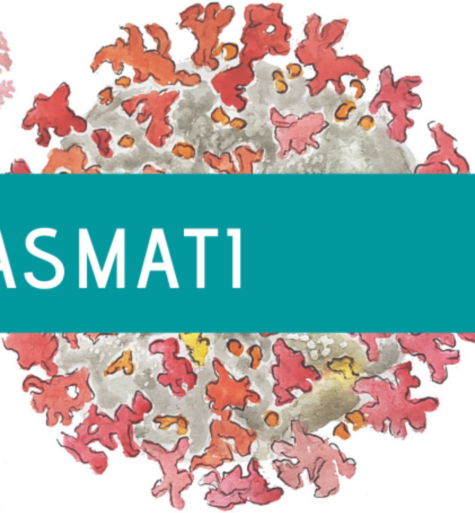

#ENTUSIASMATI

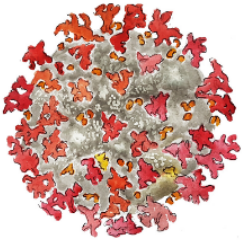

L'emergenza coronavirus ci ha messi a confronto con le nostre **paure**, derivanti da un "nemico" invisibile di cui conosciamo solo il nome in codice: covid-19.
Il clima che si respira a causa della diffusione del virus può portarci a compiere azioni irrazionali come fare finta che questa cosa non esista, "dimenticandoci" delle regole che siamo chiamati a rispettare per il nostro e altrui bene.

La **paura è un'emozione ancestrale legata all'istinto, del tutto naturale e comprensibile.** La paura tende a **paralizzare le nostre azioni**, a **toglierci energia** e a **renderci irrazionali**.
Esprimi le tue emozioni più profonde, dai un senso alla paura e trasformala in un'occasione per **riflettere e migliorare la tua quotidianità**.

Confrontati con chi ti è vicino, ingaggiati con i tuoi cari: questo ti farà scoprire che non sei da solo. Quando è necessario, **confrontati con un operatore della salute**.

Entusiasmati!
Come? Sostituisci i pensieri negativi con ricordi belli che danno conforto, immagini di viaggio e di momenti felici, di progetti per il futuro.

Ritrovare l'entusiasmo è fondamentale per tenere duro in un momento difficile e per avere tutte le carte in regola per poter ripartire alla grande quando il peggio sarà passato.

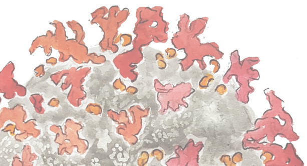

#IOSONOENGAGED

UNIVERSITÀ CATTOLICA del Sacro Cuore

EngageMinds HUB

Consumer, Food & Health Engagement Research Center

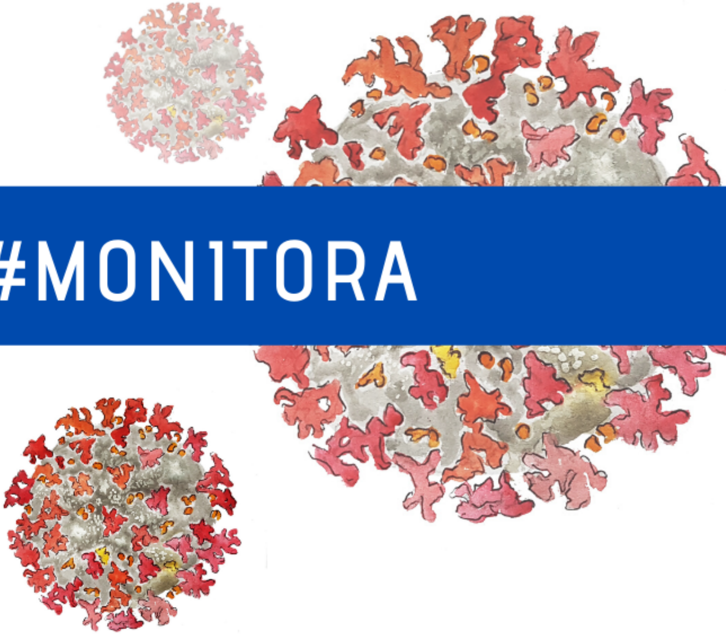

#MONITORA

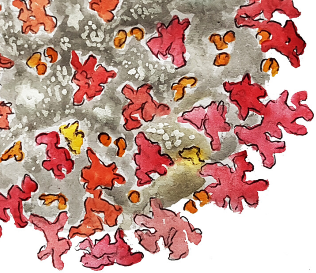

UNIVERSITÀ CATTOLICA del Sacro Cuore

EngageMinds HUB

Consumer, Food & Health Engagement Research Center

È di fondamentale importanza in un momento di emergenza provare a **monitorarsi**, ascoltando i **segnali del proprio corpo** e della **propria mente**. È anche importante imparare a decodificarli e a comunicarli.

Non solo diventare consapevoli di eventuali sintomi o malessere, ma soprattutto diventare consapevoli delle proprie azioni e delle ricadute che queste hanno sulla nostra salute e la sua promozione: pensa alla tua dieta, ai movimenti che fai nella giornata, alle cose che ti fanno stare bene o che ti rattristano.

Cerca di **riprogrammare la tua quotidianità sulla base dell'ascolto e della conoscenza che fai di te stesso**: il punto è rendersi conto che si può diventare **protagonisti della propria salute**, prendendosene cura ogni giorno in ogni singola scelta del nostro stile di vita.

#IOSONOENGAGED

UNIVERSITÀ CATTOLICA del Sacro Cuore

EngageMinds HUB

Consumer, Food & Health Engagement Research Center

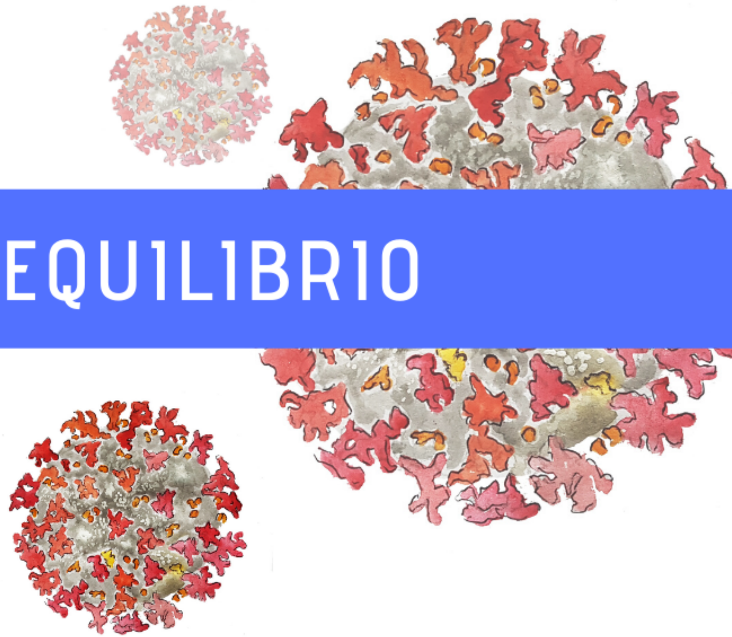

#EQUILIBRIO

Nelle situazioni di "fatica" diventa ancora più importante trovare un **equilibrio**: tra lo spavento per la diffusione del virus e la fiducia nella possibilità di farcela a tornare alla vita di prima; tra la voglia di informarsi e di capire cosa sta succedendo… e la capacità di attendere, di tollerare l'incertezza che caratterizza questi momenti in cui nessuno ha (ancora) le risposte definitive; tra i diversi ruoli che caratterizzano la nostra vita e che ne costituiscono la linfa vitale… seppure con tempi, luoghi e difficoltà nuove, **mantenere vivo il nostro impegno nelle diverse attività che ci stanno a cuore (lavorative e non, private, di volontariato)** sperimentando forme a distanza di **coinvolgimento** e di **contributo**.

Questo significa **coltivare le sfaccettature della nostra persona**, delle **nostre competenze e dei nostri talenti che si realizzano nelle relazioni famigliari, nel lavoro, nelle amicizie,** seppur in uno spazio e in un tempo "diversi" e tutti da sperimentare.

Essere in equilibrio e bilanciati in queste sfere dell'emotività e della nostra vita può facilitarci nel diventare **protagonisti della nostra salute e "engaged" rispetto ai comportamenti preventivi richiesti dalla situazione**.

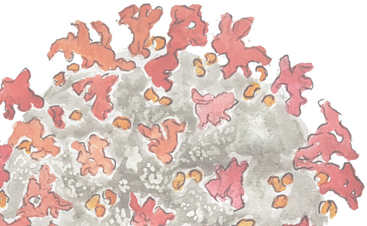

#IOSONOENGAGED

UNIVERSITÀ CATTOLICA del Sacro Cuore

EngageMinds HUB

Consumer, Food & Health Engagement Research Center

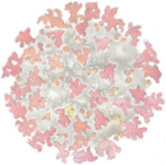

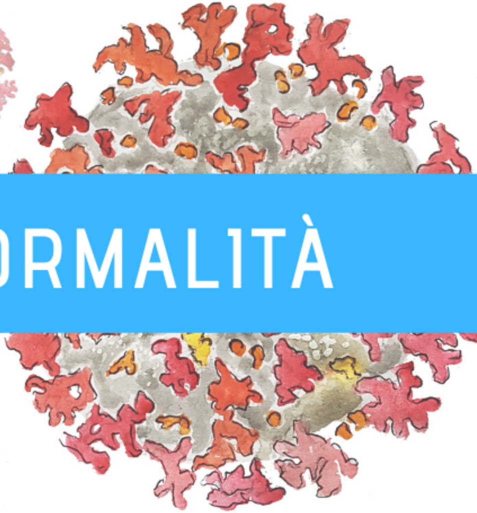

#NUOVA NORMALITÀ

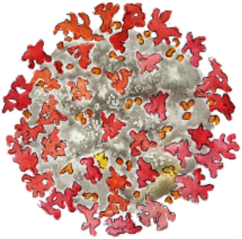

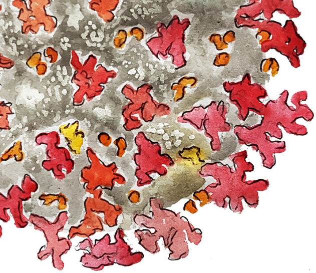

Anche in una situazione difficile come quella attuale, si possono trovare nuove routine che ci rassicurino e che ci permettano di sentire che la nostra vita può proseguire sebbene con una nuova veste.

Recuperare una **nuova forma di normalità** è possibile ed è prezioso - oggi più che mai - per affrontare e adattarsi a questo periodo nel miglior modo possibile.

Abitudini sane che prima non avevamo il tempo di mettere in pratica - come l'attività fisica in casa, la ginnastica dolce per gli anziani, la lettura di libri, contattare vecchi amici, corsi e tutorial per la crescita personale - **possono essere molto importanti per scoprire una nuova forma di normalità**.

Provare a mantenere le **abitudini della vita privata** "prima dell'emergenza" (seppur riconfigurandole nella necessità di stare confinati a casa!!) è importante per **alimentare la nostra motivazione** a tenere duro, ad aderire alle misure preventive imposte in questo momento e ad avere **maggiore positività e speranza.**

UNIVERSITÀ CATTOLICA del Sacro Cuore

EngageMinds HUB

Consumer, Food & Health Engagement Research Center

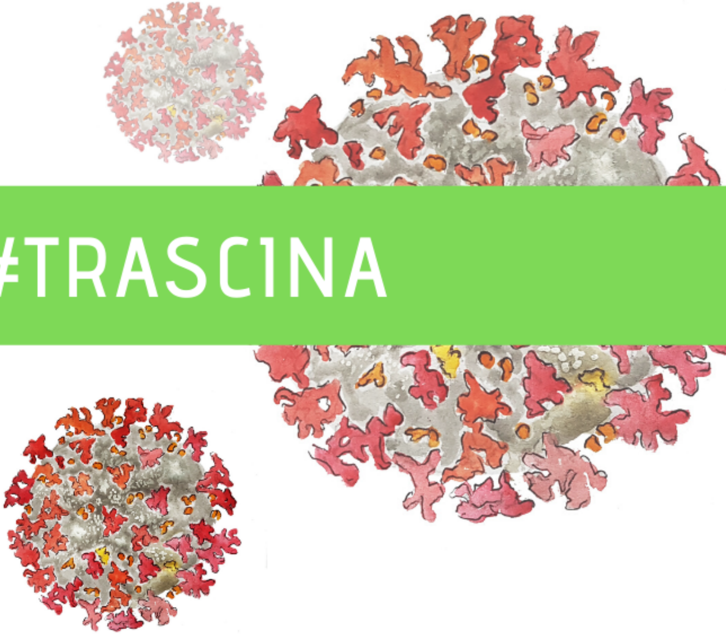

#TRASCINA

UNIVERSITÀ CATTOLICA del Sacro Cuore

EngageMinds HUB

Consumer, Food & Health Engagement Research Center

Diventare ambasciatore di **messaggi positivi** e di **buone pratiche di prevenzione** è fondamentale nel momento in cui per "fare la cosa giusta" le persone hanno bisogno di esempi virtuosi da seguire.

Essere ingaggiati significa anche questo:
mostrare agli altri che ce la si può fare!

"Io sono engaged" è il messaggio da fare proprio e da condividere nella propria rete sociale per diffondere questo modo nuovo di approcciarsi alla salute e alla propria prevenzione.

Siamo tutti chiamati a **diventare testimoni di modi nuovi, responsabili ed efficaci di rapportarci con il sistema sanitario e le sue prescrizioni e di gestire la nostra salute e la sua promozione.**

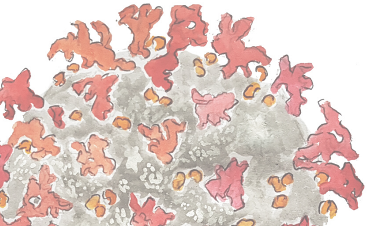

#IOSONOENGAGED

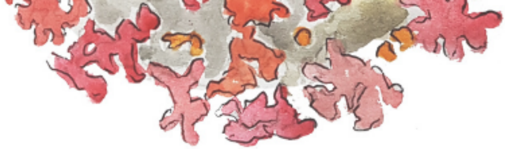

Empatizza

Naviga le giuste informazioni

Gestisci lo stress

Affidati al Sistema Sanitario

Gustati il tempo

Entusiasmati

Monitora

Equilibrio

Nuova normalità

Trascina

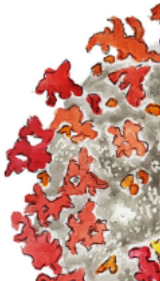

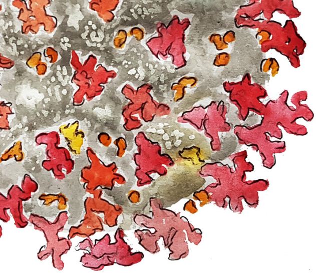

UNIVERSITÀ CATTOLICA del Sacro Cuore

EngageMinds HUB

Consumer, Food & Health Engagement Research Center

In questi giorni tenere duro e non farsi abbattere dalla preoccupazione per la grave situazione sanitaria che stiamo vivendo è faticoso.
Così come è faticoso aderire alle prescrizioni sanitarie che riconfigurano le nostre abitudini di vita quotidiana.

EngageMinds HUB - Centro di Ricerca Universitario da sempre impegnato nello studio e nella promozione delle buone condotte di salute - non si tira indietro e vuole dare un contributo ai cittadini condividendo i suoi strumenti e il suo capitale **di evidenze scientifiche sul tema dell'ENGAGEMENT.**

ENGAGEMENT come capacità di assumere un ruolo proattivo e collaborativo nel processo preventivo e di gestione dell'emergenza.
ENGAGEMENT è la parola attorno a cui ruotano le riflessioni che EngageMinds HUB vi propone nel suo vademecum.

Queste parole chiave suggeriscono spunti e appunti per vivere meglio i giorni di quarantena. Il vademecum offre qualche semplice regola per riprogettare la vita quotidiana da vero protagonista del cambiamento, diventando un buon esempio per chi ci sta accanto.

#IOSONOENGAGED

IN COLLABORAZIONE CON

Associazione Diabetici della Provincia di Milano

A.Fa.D.O.C.
ASSOCIAZIONE FAMIGLIE DI SOGGETTI CON DEFICIT DELL'ORMONE DELLA CRESCITA E ALTRE PATOLOGIE RARE

aimac
associazione italiana malati di cancro, parenti e amici

ALAMA APS
Associazione Liberi dall'Asma, dalle Malattie Allergiche, Atopiche, Respiratorie e Rare

ALOMAR ODV
ASSOCIAZIONE LOMBARDA MALATI REUMATICI

AMaR Piemonte Onlus
Associazione Malati Reumatici del Piemonte

AMaR Piemonte Young

AMICI ONLUS
Associazione Nazionale

ANMAR
Associazione Nazionale Malati Reumatici Onlus

Associazione Nazionale "Gli Amici per la Pelle"
ANAP Onlus
A sostegno dei malati di psoriasi, vitiligine ed altre patologie dermatologiche croniche

APMARR
Associazione Nazionale Persone con Malattie Reumatologiche e Rare - APS

Accademia dei Pazienti
Conoscere per Decidere

F.A.V.O.
Federazione italiana delle Associazioni di Volontariato in Oncologia

#fightthestroke

FederASMA e ALLERGIE ONLUS
Federazione Italiana Pazienti

APS Associazione PaLiNUro
PAZIENTI LIBERI DALLE NEOPLASIE UROTELIALI

RESPIRIAMO INSIEME

SALUTE Donna ONLUS

SALUTE Uomo

TUTOR
ASSOCIAZIONE TUMORI TORACICI RARI

COVID 19

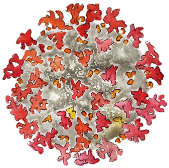

Centro di Ricerca

UNIVERSITÀ CATTOLICA del Sacro Cuore
EngageMinds HUB
Consumer, Food & Health Engagement Research Center

Direttore
GUENDALINA GRAFFIGNA

Coordinatore Area Health
SERENA BARELLO

Coordinatore Area Food
MARIAROSARIA SAVARESE

Project Manager
CATERINA BOSIO

Illustrazioni e Progetto Grafico
MARINA BARELLO

Con la collaborazione delle Associazioni di Pazienti: ADPMi | A.FA,D.O.C. | AIMAC | ALAMA | ALOMAR | AMaR | AMICI | ANMAR | ANAP | APMARR | EUPATI | F.A.V.O. | FEDERASMA | FightTheStroke | PALINURO | RESPIRIAMO INSIEME | SALUTE DONNA | SALUTE UOMO | TUTOR

UNIVERSITÀ CATTOLICA del Sacro Cuore

EngageMinds HUB

Consumer, Food & Health Engagement Research Center

CONTATTI

Sito Internet

www.engagemindshub.com

Pagina Facebook

@EngageMindsHUB

Pagina Linkedin

@EngageMinds HUB Research Center

#IOSONOENGAGED
